# Enriched Au nanoclusters with mesoporous silica nanoparticles for improved fluorescence/computed tomography dual‐modal imaging

**DOI:** 10.1111/cpr.13008

**Published:** 2021-02-25

**Authors:** Yifang Yuan, Ronghui Zhou, Ting Li, Shuang Qu, Hua Bai, Jiawu Liang, Xiaoxiao Cai, Bin Guo

**Affiliations:** ^1^ Department of Stomatology Chinese PLA General Hospital Beijing China; ^2^ State Key Laboratory of Oral Diseases National Clinical Research Center for Oral Diseases West China Hospital of Stomatology Sichuan University Chengdu China

**Keywords:** Au nanoclusters, computed tomography, dual‐modal imaging, fluorescence, mesoporous silica nanoparticles

## Abstract

**Objectives:**

Au nanoclusters (AuNCs) have been used widely in fluorescence bio‐imaging because of their good fluorescence, small particle size and non‐cytotoxicity. AuNCs are also efficient in computed tomography (CT) imaging. Hence, a dual‐modal imaging probe can be constructed without any complicated modification processes by exploiting the excellent performance of AuNCs. In the present study, AuNCs were enriched with mesoporous silica nanoparticles (MSNs) to obtain enhanced fluorescence/CT dual‐modal imaging, which was capable of acquiring more imaging information for diseases compared with single‐mode imaging.

**Materials and methods:**

Biocompatible bovine serum albumin (BSA)‐capped AuNCs were prepared and loaded into amine‐functionalized MSNs to form MSN@AuNCs. BSA‐AuNCs, MSNs, and MSN@AuNCs were characterized by ultraviolet‐visible (UV‐vis) spectra, transmission electron microscopy (TEM), fluorescence spectra, and zeta potential. CT imaging was recorded using micro‐CT scanning. Fluorescence imaging was measured using confocal laser scanning microscopy and flow cytometry.

**Results:**

The prepared AuNCs and MSNs possessed good properties as previously reported. The fluorescence intensity and CT value of the AuNCs were enhanced after being enriched with MSNs. The nanoparticles were both non‐cytotoxic. Confocal laser scanning microscopy and flow cytometry indicated that MSN@AuNCs in CAL‐27 cells showed improved fluorescence imaging compared with simple AuNCs at the same concentration.

**Conclusions:**

The results revealed that the strategy of enriching AuNCs with MSNs can obtain highly sensitive fluorescence/CT dual‐modal imaging, which indicated the potential of this nanoparticle in the diagnosis and treatment of disease.

## Graphical abstract

BSA‐functionalized AuNCs were synthesized in one step and then enriched into the pores of biocompatible MSNs through electrostatic interactions and carboxy‐amine bonds. The local concentration of AuNCs was increased apparently, and both fluorescence and CT imaging were improved simultaneously

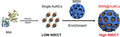

## 1 INTRODUCTION

Near‐infrared (NIR) fluorescence imaging has shown great potential in the field of biological imaging because of its advantages, such as high imaging sensitivity and strong operability.^1^, ^2^, ^3^ However, the further application of NIR fluorescence imaging probes to obtain high brightness is limited by concentration requirements and low luminous efficiency. Moreover, fluorescence imaging lacks high spatial resolution, which adversely affects the identification of anatomical information. Computed tomography (CT) is an approved diagnostic tool that is able to obtain abundant anatomical information; however, it has relatively low sensitivity.^4^, ^5^, ^6^ Dual‐modal imaging, integrating two imaging methods, can provide complementary information and thus might satisfy the requirements for accurate diagnosis of diseases compared with single‐modal imaging.^7^, ^8^, ^9^ To construct fluorescent/CT dual‐modal imaging probes, two imaging techniques are required to realize in one imaging reagent. Currently, dual‐modal imaging probes based on NIR fluorescence usually use nanoparticles as carriers, and different agents are capped with the nanoparticles through physical or chemical approaches.^10^, ^11^, ^12^ Generally, the nanoparticle carriers include liposomes, dendrimers, polymer micelles, carbon nanotubes and SiO_2_.^13^, ^14^ With the fluorescence and CT imaging reagents loaded on, the modified nanoparticles are then used for dual‐modal imaging. Nevertheless, the conjugation of different media requires complex processes such as synthesis, modification, assembly and purification, which will inevitably cause various deficiencies, such as an increase in the particle size, poor dispersion and a decrease in the respective imaging effect.^15^ Hence, it is important to develop a simple and high brightness dual‐modal imaging probe for imaging analysis.

Among numerous fluorescence agents, gold nanoclusters (AuNCs) are acknowledged for their excellent NIR fluorescence performance and low cytotoxicity.^16^, ^17^ Furthermore, AuNCs possess CT imaging efficiency, which makes them suitable as CT contrast reagents.^18^, ^19^, ^20^ Therefore, AuNCs can be used directly as fluorescence/CT dual‐modal imaging probes. For instance, Mao's group reported the application of AuNCs in fluorescence/CT dual‐modal imaging for tumour localization.^21^ However, imaging probes based on simple AuNCs exhibited low luminous efficiency and poor imaging sensitivity in vitro and in vivo.^22^ Furthermore, the quantum yield of AuNCs was only 6.85% according to our previous study. As an exogenous nanomaterial, the high concentration required to improve imaging efficiency might lead aggregation‐induced quenching and bring a certain level of toxicity. Hence, it remains challenging to develop an enhanced fluorescence and CT dual‐modal imaging probe. Specially, it is feasible to improve the imaging sensitivity of fluorescence by increasing the concentration of the AuNCs without aggregation‐caused quenching because of the large Stokes shift of the red‐emitting AuNCs.^23^, ^24^ Recently, the specific structure and uniform pores of biocompatible mesoporous silica nanoparticles (MSNs) have led to their approval as an enriching contrast medium to obtain increased concentrations of nanoparticles.^25^, ^26^, ^27^ Zhou et al have developed an enriched strategy to assemble Mn‐doped ZnSe quantum dots with MSNs to improve fluorescent and magnetic resonance (MRI) imaging.^15^ Therefore, it is reasonable to anticipate that high fluorescence/CT dual‐modal bio‐imaging could be realized by assembling AuNCs with MSNs.

**SCHEME 1:**
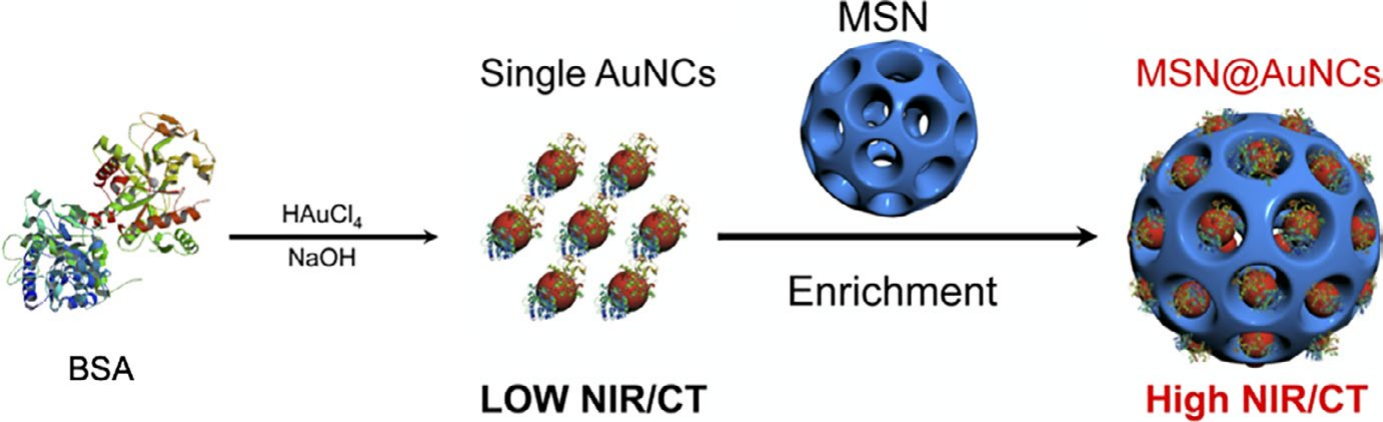
Schematic of enhanced fluorescence/CT dual‐modal imaging process

Bovine serum albumin (BSA), a type of qualified protein modifier, can be capped with AuNCs to supply extra properties such as reducing and stabilizing the nanoparticles.^28^, ^29^ Besides, BSA is widely used as a coating material in drug delivery because of its high biocompatibility and simple capping procedure.^30^, ^31^ In the present study, BSA‐functionalized AuNCs were synthesized in one step and then enriched into the pores of biocompatible MSNs through electrostatic interactions and carboxy‐amine bonds (Scheme 1). The local concentration of AuNCs was increased apparently, and both fluorescence and CT imaging were improved simultaneously. To compare the fluorescence and CT imaging sensitivity of enriched nanoparticles with that of simple AuNCs, the nanoparticles were characterized using physical and chemical measurements, and in vitro experiments.

## 2 MATERIALS AND METHODS

### 2.1 Materials

Chloroauric acid (HAuCl_4_), triethylene glycol alcohol (TEA), tetraethoxysilane (TEOS) and 3‐aminopropyl triethoxysilane (APTS) were purchased from Aladdin Co., Ltd. (Shanghai, China). Bovine serum albumin (BSA) was supplied from BioFroxx (Einhausen, Germany). Sodium hydroxide (NaOH), ethanol and cyclohexane were obtained from Kelong Chemical Co., Ltd. (Chengdu, China). 1‐ethyl‐3‐(3‐dimethylaminopropyl) carbodiimide (EDC), N‐hydroxysuccinimide (NHS) and cetyltrimethylammonium chloride (CTAC) were provided by J & K Scientific Ltd. Dulbecco's modified Eagle medium (DMED/High Glucose), Roswell Park Memorial Institute (RPMI) 1640 medium, foetal bovine serum (FBS), penicillin‐streptomycin, 0.25% trypsin‐EDTA and phosphate‐buffered saline (PBS) were purchased from GE Healthcare Life Sciences. The Cell counting Kit‐8 (CCK‐8) was provided from Dojindo Laboratory. 4,6‐diamino‐2‐phenylindole (DAPI) was supplied from Sigma‐Aldrich Chemical Co., Ltd.

### 2.2 Preparation of BSA‐capped AuNCs

Following a previously method reported by Ding's group, the BSA‐capped AuNCs were prepared by a simple process.^32^ Briefly, HAuCl_4_ (5 mL, 10 mM) and BSA (5 mL, 50 mg/mL) were mixed and stirred quickly at 37 ℃ for 2 minutes. NaOH solution (0.5 mL, 1 M) was subsequently added to adjust the pH value. The reaction mixture was kept under vigorous stirring at 37 ℃ for 12 hours. Finally, a dark brown solution was obtained. The BSA‐functionalized AuNCs solution was maintained at 4℃ for further experimentation.

### 2.3 Preparation of NH2‐MSNs

According to a previously reported approach, amine‐functionalized MSNs were prepared based on using cationic surfactant CTAC and TEA as the template.^24^ The CTAC solution (48 mL, 25 wt%) and TEA (0.36 g) were added simultaneously into 72 mL of distilled water and rigorously stirred at 60 ℃ for 1 hour. Then, TEOS in cyclohexane (40 mL, 5 v/v%) was slowly dropped into the reaction system and incubated under gentle stirring for another 12 hours. The obtained white jelly was allowed to cool to room temperature naturally before being washed with ethanol (10 864 × *g*, 10 minutes) for several times. The resultant MSNs were dispersed in NH_4_NO_3_ solution and then refluxed at 60°C for 6 hours twice to remove the template. Finally, the white precipitates were washed with ethanol (10 864 × *g*, 10 minutes) twice.

To gain amine‐functionalization MSNs, 0.1 mL APTS was dropped into an MSN ethanol solution and refluxed at 78°C for 12 hours under gentle stirring. After that, to purify the products, they were centrifuged three times at 10 864 × *g* for 10 minutes and finally dispersed in distilled water. The NH_2_‐MSNs were stored in 4°C for further use.

### 2.4 Preparation of MSN@AuNCs

Typically, 1 mL BSA‐AuNCs solution was first reacted with EDC (2 mg) and NHS (1 mg). After a few minutes, 1 mL of NH_2_ − MSNs (10 mg/mL) was added into the reaction system, which was further stirred at room temperature for 4 hours. The obtained MSN@AuNCs were washed three times by centrifugation (10 864 × *g*, 10 minutes) using distilled water to remove the supernatant and then re‐dispersed with distilled water. The resultant products were store at 4°C.

### 2.5 Characterizations of AuNCs, MSNs and MSN@AuNCs

The particle sizes and zeta potential were measured using a nanoparticle analyser SZ‐100 (Horiba Scientific, Kyoto, Japan). Inductively coupled plasma atomic emission spectroscopy (ICP‐OES) was used to analyse the quantities of AuNCs on the MSN@AuNCs and to determine the concentrations of the two nanoparticles. Ultraviolet‐visible (UV‐vis) absorption spectra were assessed on a Mettler Toledo UV5Nano (Mettler‐Toledo Ltd., Leicester, UK). The morphology and compositional distribution of AuNCs, MSNs and MSN@AuNCs were carried out using a JEM‐2100PLUS Transmission Electron Microscope (TEM). The fluorescence spectrophotometer (Shimadzu RF‐5301PC, Tokyo, Japan) was used to record the fluorescence spectra and the fluorescence decay behaviour of AuNCs and MSN@AuNCs. The fluorescence stability of AuNCs, MSN@AuNCs and Rhodamine were also assessed using the fluorescence spectrophotometer.

### 2.6 CT imaging in vitro

AuNCs and MSN@AuNCs at a concentration range of 0‐5 mM were poured into 200 μL tubes and analysed using micro‐CT scanning (μCT, SCANCO Medical AG, Wangen‐Brüttisellen, Switzerland) to examine the X‐ray absorption property of AuNCs. The CT scanning parameters were as follows: voxel size, 34.4 μm; energy/intensity, 70 kV, 200 μA, 14 W; field of view, 34 mm × 110 mm; and integration time, 1000 ms CT images were reconstructed according to the recorded data, and the CT value (Hounsfield Units, HU) was acquired using SCANCO Evaluation.

### 2.7 Cell culture

The mouse fibroblast cell line (L929), human tongue squamous cell carcinoma cell line (CAL‐27), human adenoid cystic carcinoma cell line (ACC‐2) and human oral squamous carcinoma cell line (SCC‐25) were obtained from the Key Laboratory of Oral Diseases of Sichuan University (Chengdu, China). CAL‐27 cells and SCC‐25 cells were maintained at a humidified incubator (37°C, 5% CO_2_) respectively in DMEM/High Glucose and DMEM/F12 supplemented with 10% FBS and 1% penicillin‐streptomycin. L929 cells and ACC‐2 cells were maintained in a humidified incubator (37°C, 5% CO_2_) in RPMI 1640 supplemented with 10% FBS and 1% penicillin‐streptomycin. The medium was renewed every two days.

### 2.8 Cell cytotoxicity of AuNCs and MSN@AuNCs

When the cells had proliferated to 80%, they were harvested for experimentation. Cell cytotoxicity was measured using the CCK‐8 assay. Briefly, L929 cells, CAL‐27 cells, ACC‐2 cells and SCC‐25 cells were counted and seeded in 96‐well plates at a density of 10^4^ cells per well, which contained 100 μL of complete medium. Then, the cells were cultured in a 37°C incubator with 5% CO_2_ overnight to adhere. Afterwards, the cells were washed with PBS, and new complete medium with various concentrations of AuNCs and MSN@AuNCs (calculated by the concentration of AuNCs) from 100 to 800 nM was added into wells. Five wells for each condition were set up as positive controls. Then, the cells were incubated for another 48 hours. After exposure, the medium was removed, and cells were washed with PBS. Subsequently, 100 μL FBS‐free medium with 10% CCK‐8 was added to wells. After approximately 2 hours, the medium turned to yellow or brown. The cell cytotoxicity of AuNCs and MSN@AuNCs was analysed by detecting the absorbance at 450 nm with a microplate reader (Thermo Scientific, Waltham, MA, USA).

### 2.9 Fluorescence imaging and intensity of cells in vitro

CAL‐27 cells and L929 cells were selected for fluorescence imaging experiment. The cells were seeded in several 35 mm confocal dishes at 10^5^ cells per dish with 2 mL of complete medium and cultured overnight to adhere. When the cells had proliferated appropriately, AuNCs and MSN@AuNCs were dispersed into the medium, making sure the concentration of AuNCs 1 μM every dish, and then re‐incubated with cells for 6 hours. Next, the cells were rinsed using PBS three times and then fixed with 1 mL 4% paraformaldehyde for 25 minutes. Then, the fixator was removed, and the cells were washed using PBS three times (5 minutes each). The cells were subsequently incubated with 1 μg mL^‐1^ DAPI/PBS solution for 10 minutes to stain the cell nuclei. The cells were mounted with 10% glycerol and observed under a confocal laser scanning microscopy (Olympus Fluoview FV3000, Tokyo, Japan). DAPI staining was recorded under an excitation wavelength of 405 nm and emission wavelengths of 440‐470 nm, while the cell uptake of AuNCs was detected under an excitation wavelength of 488 nm and emission wavelengths of 550‐650 nm.

The quantitative analysis of fluorescence intensity was carried out using flow cytometry. CAL‐27 cells and L929 cells were counted and seeded in 6‐well plates at a density of 10^5^ cells per well, and incubated with 2 mL of complete medium in a 37°C incubator. When the cells had adhered sufficiently, the medium was removed, and new medium with 1 μM AuNCs and MSN@AuNCs (calculated by AuNCs) was added and incubated with the cells for 12 hours and 16 hours. Afterwards, the cells were digested with 0.25% trypsin‐EDTA, and washed twice with PBS to removing redundant media and materials. The collected cells were re‐dispersed in 500 μL PBS for flow cytometry analysis (FACS Aria II; BD Biosciences, San Jose, CA, USA). The fluorescence intensity of cells was obtained between 550 nm and 650 nm under an excitation wavelength of 355 nm.

### 2.10 Statistical analysis

The experiments in this study were all repeated at least three times under the same conditions. All data were analysed using an independent t test or one‐way ANOVA using SPSS 26.0, and a P value < 0.05 was considered statistically significant.

## 3 RESULTS

### 3.1 Preparation and characterization of AuNCs, MSNs and MSN@AuNCs

**FIGURE 1:**
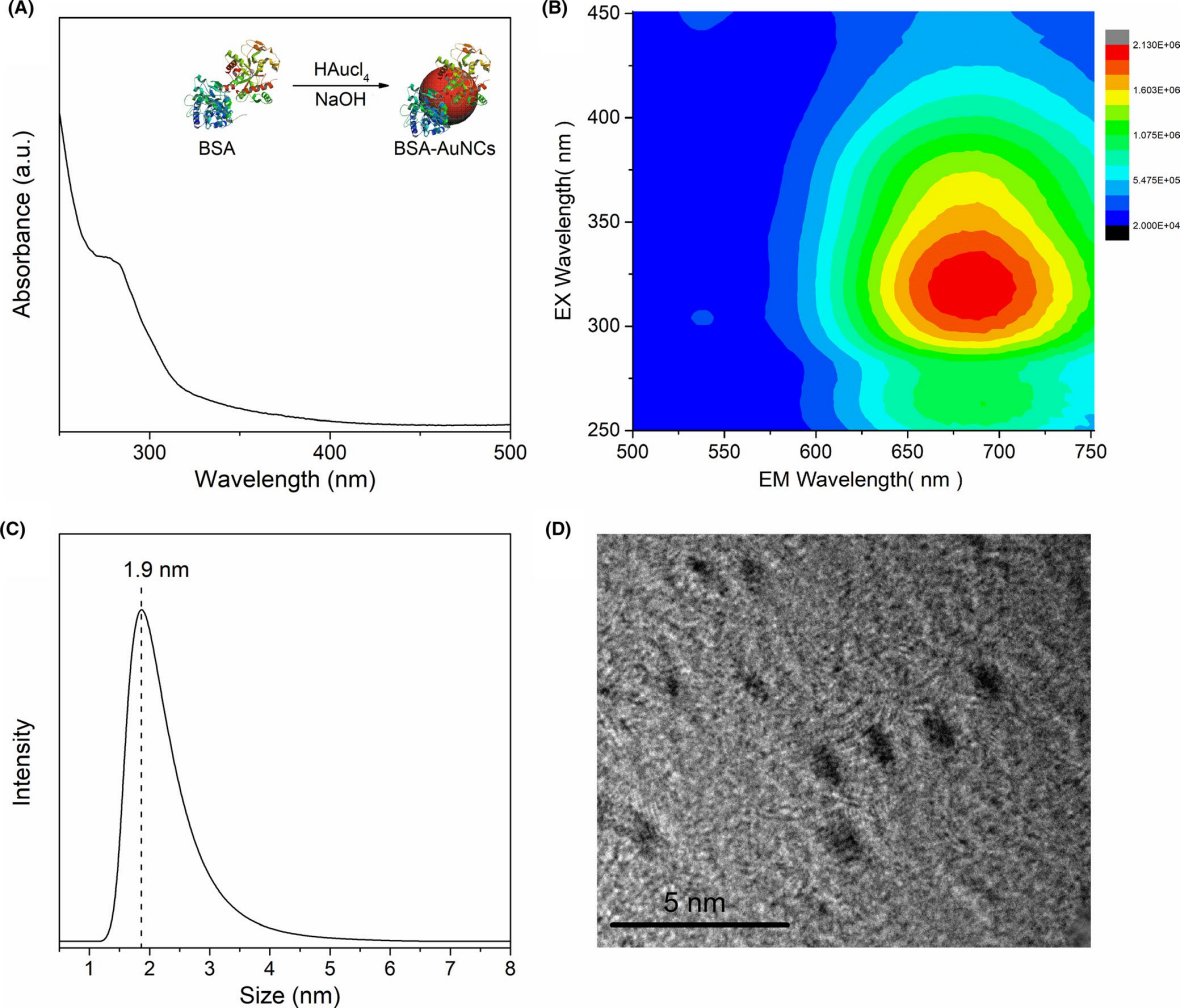
Characterizations of BSA‐AuNCs: A, UV‐vis absorbance spectroscopy. Inset: preparation of BSA‐AuNCs; B, 3D fluorescence spectroscopy; C, particles size; and D, TEM image. Abbreviations: bovine serum albumin (BSA), Au nanoclusters (AuNCs), ultraviolet‐visible (UV‐vis), transmission electron microscopy (TEM)

Based on AuNCs and MSNs, a highly sensitive fluorescence/CT dual‐modal imaging probe was constructed through enrichment, without complicated modification and assembly, aiming to realize enhanced dual‐modal bio‐imaging. AuNCs functionalized with BSA were synthesized by a simple method (Figure 1A inset). The BSA‐AuNCs solution appeared brown under daylight and emitted a red colour when exposed to UV light. The UV‐vis absorption spectra of BSA‐AuNCs exhibited a peak near 280 nm (Figure 1A), which corresponded with the characteristic absorption of BSA.^30^ 3D fluorescence spectra showed that the excitation and emission wavelengths of the AuNCs were both wide and the peaks of the excitation and emission wavelengths were approximately around 300‐350 nm and 600‐650 nm, respectively (Figure 1B). The average particle size of the BSA‐AuNCs was approximately 2 nm, as shown in Figure 1C. To further confirm the diameter of the nanoclusters, the morphology was obtained using TEM (Figure 1D). The images showed the lattice of Au metal and indicated that the size of the BSA‐AuNCs was approximately 2 nm.

**FIGURE 2:**
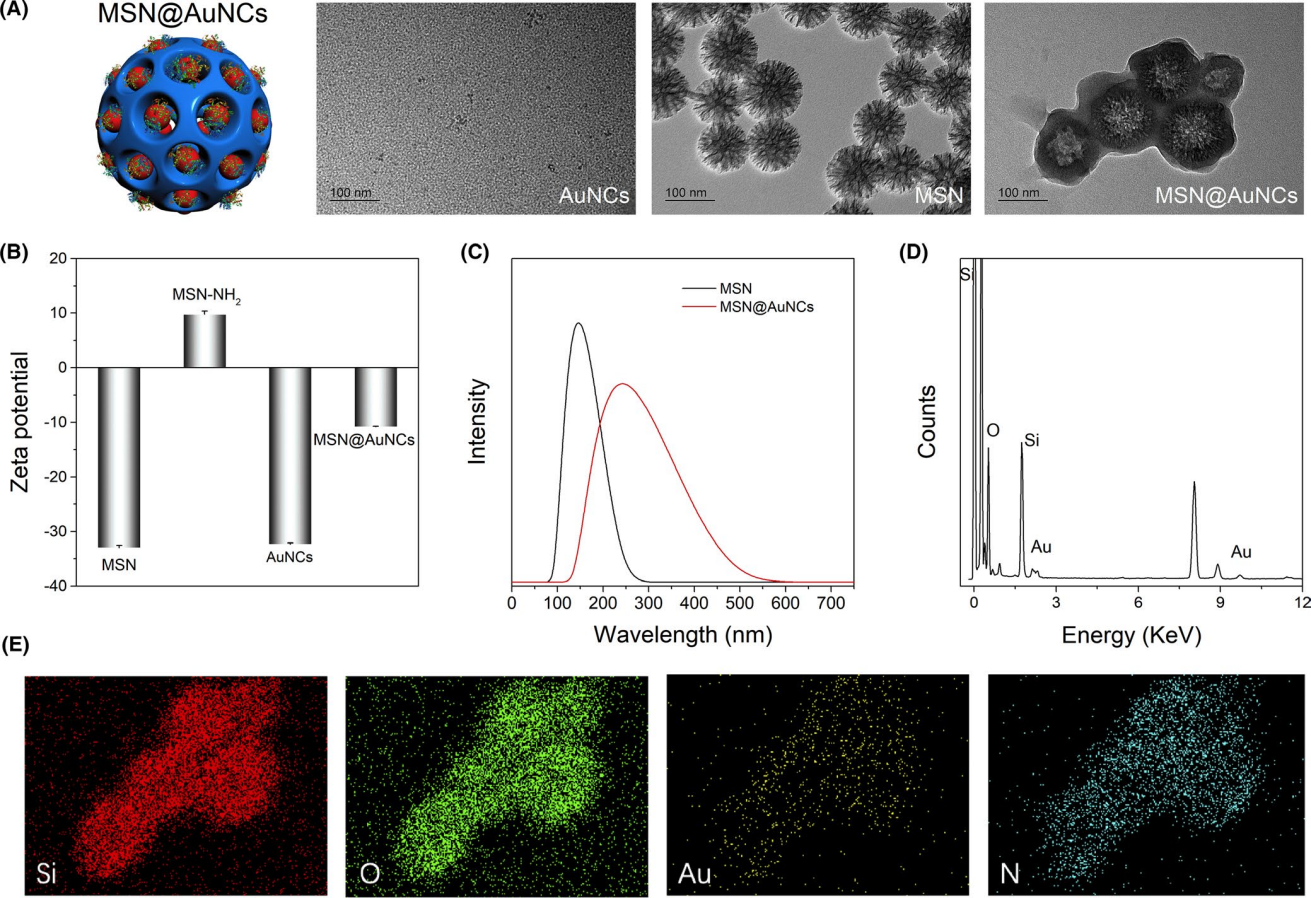
A, Schematic of MSN@AuNCs and TEM images of AuNCs, MSN and MSN@AuNCs; B, zeta potential of MSN, MSN‐NH2, AuNCs and MSN@AuNCs; C, particle size of MSN and MSN@AuNCs; D, EDX spectrum of MSN@AuNCs; and E, EDX mappings of MSN@AuNCs. Abbreviations: mesoporous silica nanoparticles (MSNs), energy dispersive X‐ray (EDX)

MSNs were prepared and amine‐functionalized. AuNCs were enriched into NH_2_‐MSNs through EDC/NHS coupling.^15^ As shown in Figure 2A, the diameter and morphology of the AuNCs, MSNs and MSN@AuNCs were detected by TEM. The AuNCs had a small size and were monodispersed in water. Uniform dendritic MSNs, with an average size of approximately 100 nm and plenty of pores larger than AuNCs, were observed. The particle size of the MSN@AuNCs was similar to that of MSNs at approximately 100 nm and the morphology remained spherical in shape, with little change. The TEM images indicated that the AuNCs were loaded into the MSNs. As shown in Figure 2B, the zeta potentials of the MSNs and AuNCs were negative, at approximately − 32.9 mV and − 32.4 mV, respectively. After amine functionalization, the charge of the MSNs became positive, at approximately 9.8 mV. Finally, the zeta potential of the resultant products was approximately − 10.8 mV after the negative AuNCs were enriched. Hence, the obvious change in the zeta potential supported the successful synthesis of MSN@AuNCs. As measured by the nanoparticle analyser, the average sizes of the MSNs and MSN@AuNCs were approximately 200 nm and 250 nm, respectively, as shown in Figure 2C. The diameter of these nanoparticles was larger than that assessed using TEM because of aggregation. Nevertheless, the dispersity of the MSN@AuNCs in solution was considered to be acceptable. To further confirm that AuNCs were enriched into MSNs, the compositional distribution of MSN@AuNCs was investigated by EDX characterization. The EDX spectrum indicated the Au signal in the MSN@AuNCs in addition to Si and O signals (Figure 2D). Moreover, the EDX elemental mapping of Si, O, Au and N was shown in Figure 2E, which further revealed the formation of MSN@AuNCs.

**FIGURE 3:**
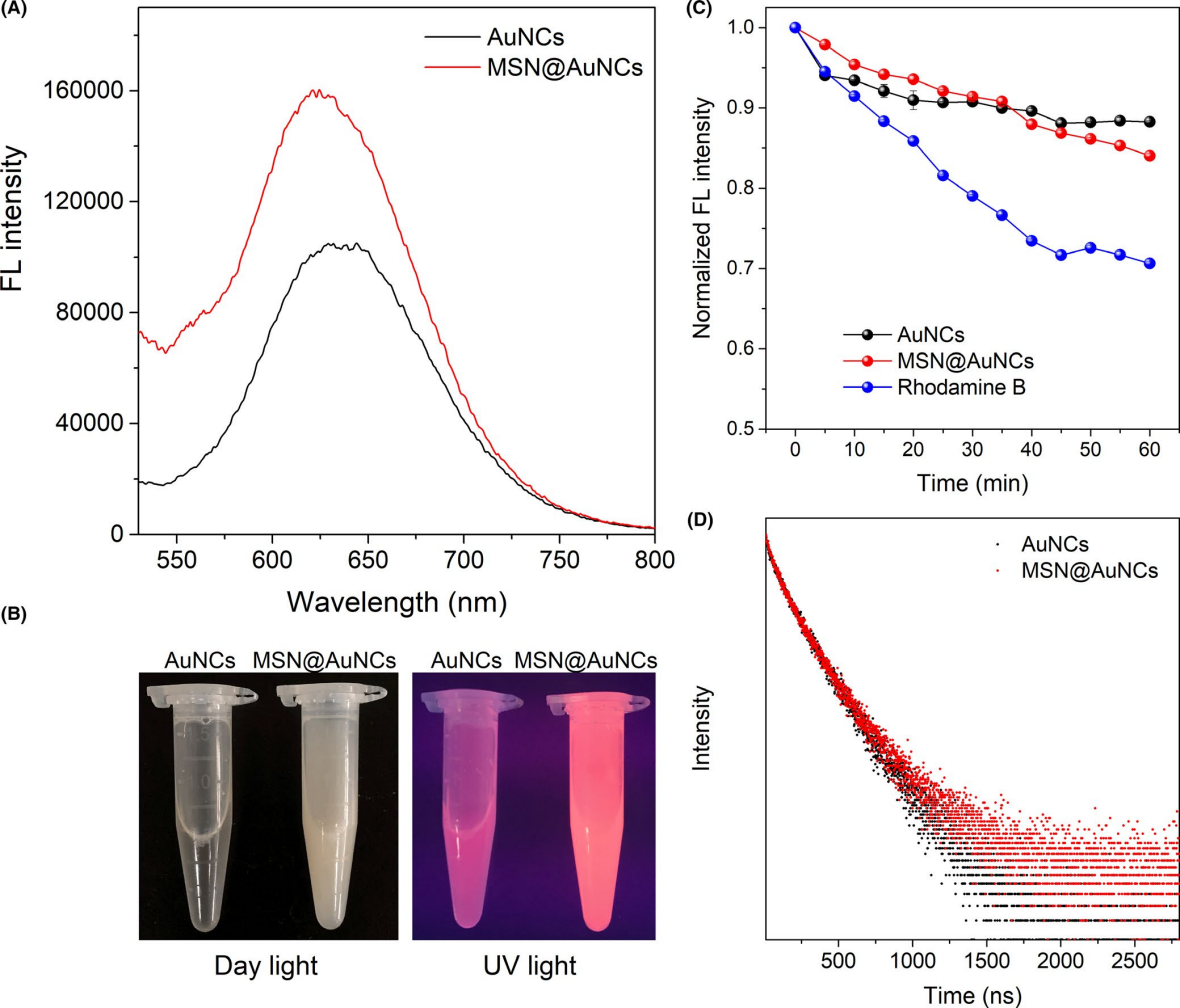
Characterizations of AuNCs and MSN@AuNCs: A, fluorescence emission spectra; B, photographs under daylight and UV light; C, fluorescence stability of AuNCs, MSN@AuNCs and Rhodamine; and D, fluorescence lifetime

To compare the fluorescence of AuNCs and MSN@AuNCs, the concentration of MSN@AuNCs and AuNCs was first adjusted to a level such that the amount of AuNCs was equal, as determined using ICP‐OES quantification. The fluorescence spectra of these nanoparticles were investigated (Figure 3A). The AuNCs and MSN@AuNCs showed emission peaks at around 630 nm and 620 nm, respectively, under a 360 nm excitation wavelength, while the fluorescence intensity of the MSN@AuNCs was distinctly higher than that of the simple AuNCs. As shown in Figure 3B, the AuNCs monodispersed in water formed a brown transparent solution under daylight, while the MSN@AuNCs were a slightly red, white colloidal substance, and when dispersed in water, the solution was opaque under daylight. The AuNCs and MSN@AuNCs solutions emitted red fluorescence under UV light, and the fluorescence of the MSN@AuNCs was much brighter than that of the simple AuNCs. To explore their fluorescence stability, AuNCs and MSN@AuNCs were excited under 360 nm wavelength persistently using a fluorescence spectrophotometer for 1 hour and the fluorescence intensity was recorded every 5 minutes. The photostability of Rhodamine was recorded as well. The fluorescence of AuNCs and MSN@AuNCs showed a slight fluctuation and decreased by approximately 10 to 15% after 1 hour of irradiation, while the fluorescence of Rhodamine declined significantly (Figure 3C). Thus, the fluorescence stability of AuNCs and MSN@AuNCs was better than that of the chemical fluorone dye. Furthermore, the fluorescence lifetime was determined by the luminescence decay curves in Figure 3D. There was no significant difference between the AuNCs and the MSN@AuNCs, which indicated that the fluorescence of the AuNCs was stable, without enrichment caused self‐absorption after assembly.

### 3.2 CT imaging compared between AuNCs and MSN@AuNCs in vitro

**FIGURE 4:**
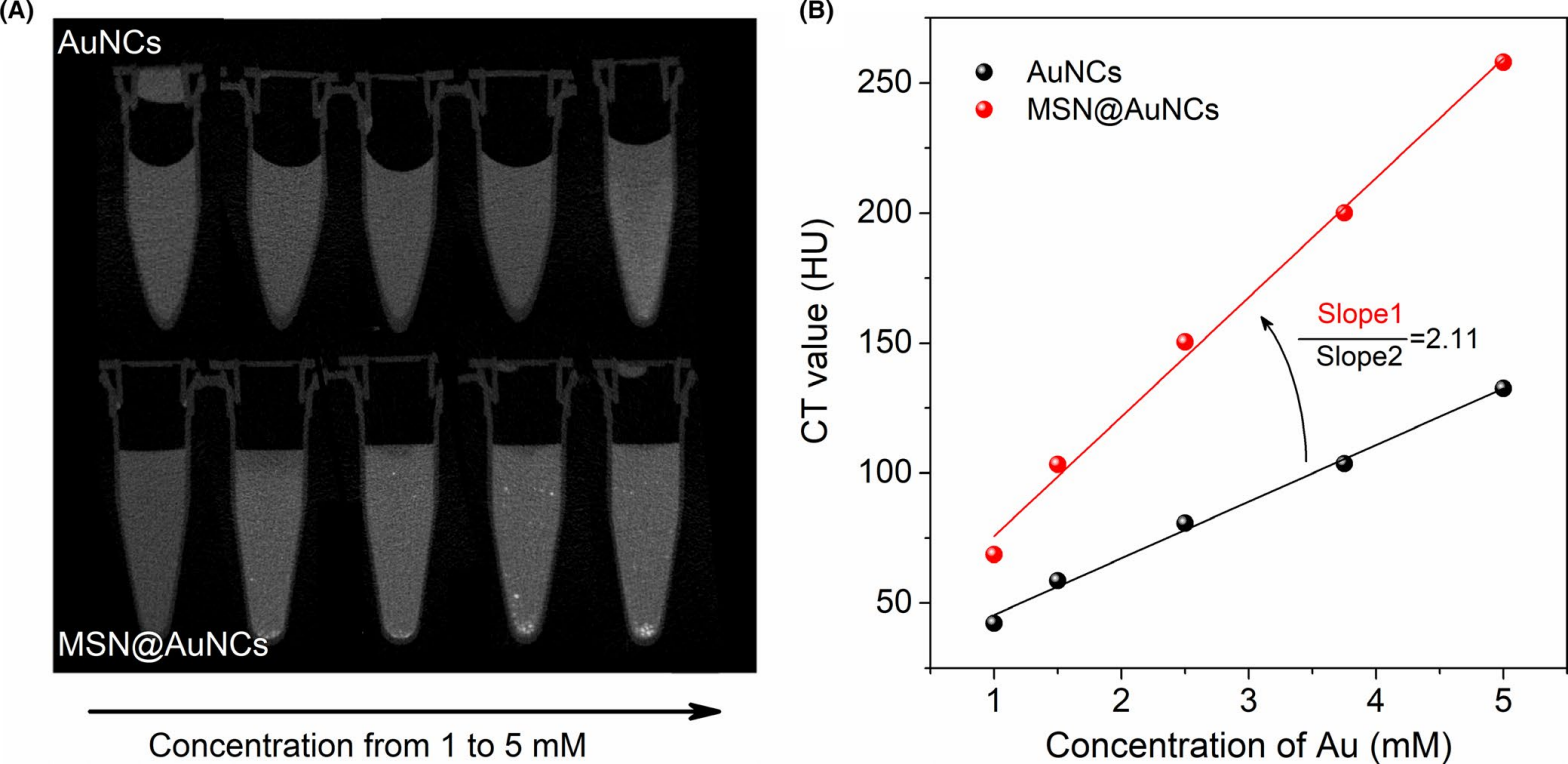
Micro‐CT detection of AuNCs and MSN@AuNCs: (A) transverse CT images in vitro and (B) X‐ray attenuation (HU) values at various concentrations. Abbreviations: computed tomography (CT), Hounsfield Units (HU)

AuNCs enriched into MSN were expected to enhance the CT intensity compared with that of simple AuNCs. The feasibility of MSN@AuNCs as a sensitive CT reagent was tested by CT scanning in vitro as shown in Figure 4. The CT intensity of both AuNCs and MSN@AuNCs continuously increased as the concentration of AuNCs increased from 1 to 5 mM. In addition, the intensity of MSN@AuNCs was obviously higher than that of the simple AuNCs at the same concentrations (Figure 4A). Moreover, several high‐density shadows, representing MSN@AuNCs aggregation and sedimentation, could be observed, which reflected the enhanced CT imaging capabilities of the MSN@AuNCs. Figure 4B showed that there was a good linear relationship between the MSN@AuNCs and the HU values, and between the AuNCs and the HU values. The slope of the MSN@AuNCs curve was 2‐fold greater than that of the AuNCs curve.

### 3.3 Cell cytotoxicity of AuNCs and MSN@AuNCs

**FIGURE 5:**
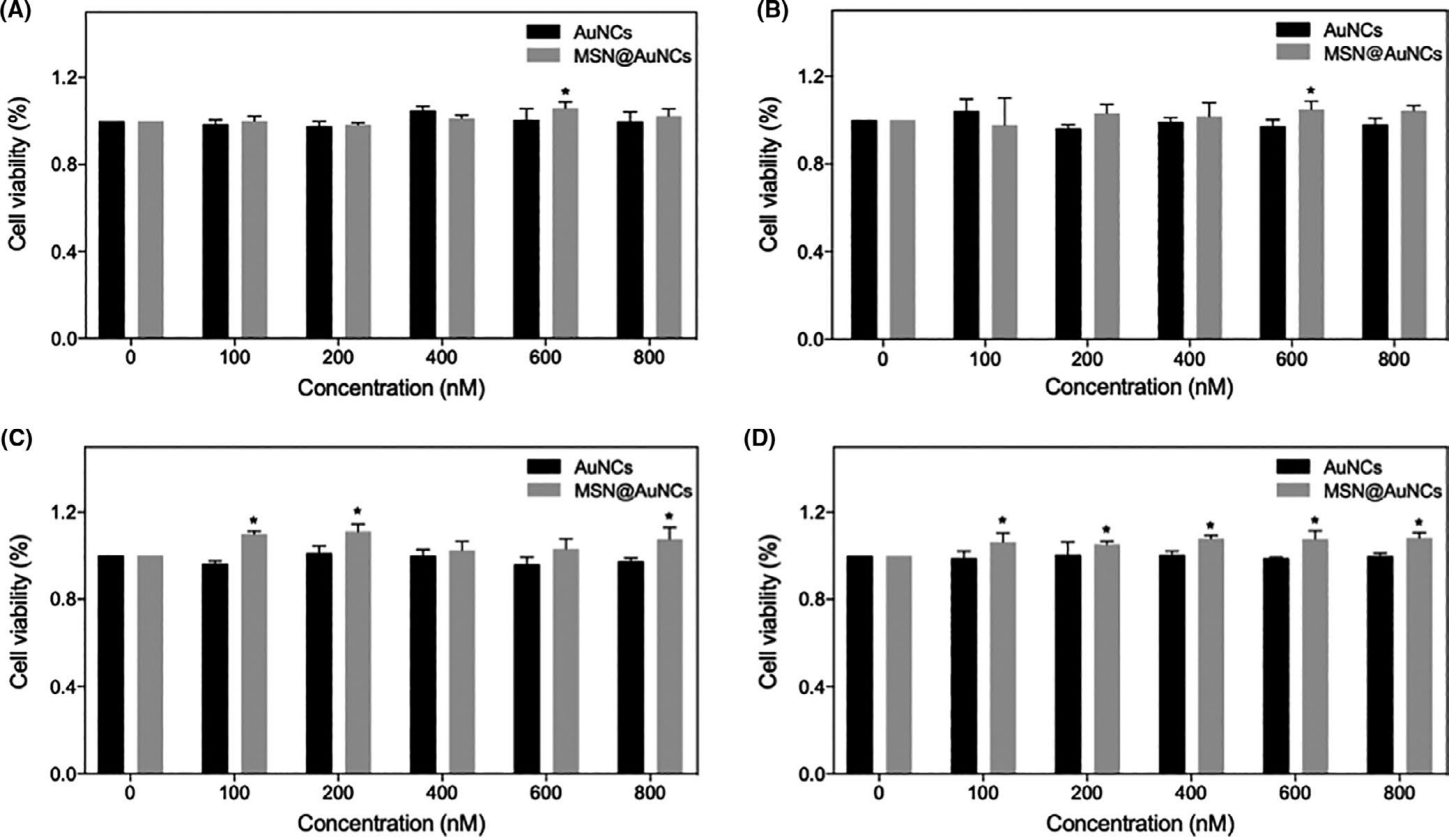
Cell viability of A, L929 cells, B, CAL‐27 cells, C, ACC‐2 cells and D, SCC‐25 cells exposed to AuNCs and MSN@AuNCs at various concentrations (100, 200, 400, 600 and 800 nM AuNCs) for 48 hours treatment. Data are presented as mean ± SD (n = 3). *P < .05

The cytotoxicity of the AuNCs and MSN@AuNCs towards L929 cells, CAL‐27 cells, ACC‐2 cells, and SCC‐25 cells was measured using the CCK‐8 assay. After cultured with AuNCs and MSN@AuNCs in a diverse concentration range of 100 to 800 nM for 48 hours, the L929 cells were not affected by any concentration, and the cell viability in 600 nM MSN@AuNCs was statistically higher than that in the control (Figure 5A). For CAL‐27 cells, there was no increase in cytotoxicity by AuNCs and MSN@AuNCs incubation after 48 hours, even at 800 nM (Figure 5B). Therefore, no significant decline in the cell viability at concentrations from 100 to 800 nM was detected after 48 hours of incubation. Furthermore, the cytotoxicity of AuNCs and MSN@AuNCs was verified in ACC‐2 cells and SCC‐25 cells, and no significant cytotoxicity was detected (Figure 5C and 5D). These results showed that the AuNCs and MSN@AuNCs at 800 nM could be considered non‐toxic.

### 3.4 Fluorescence imaging and intensity of cells in vitro

**FIGURE 6:**
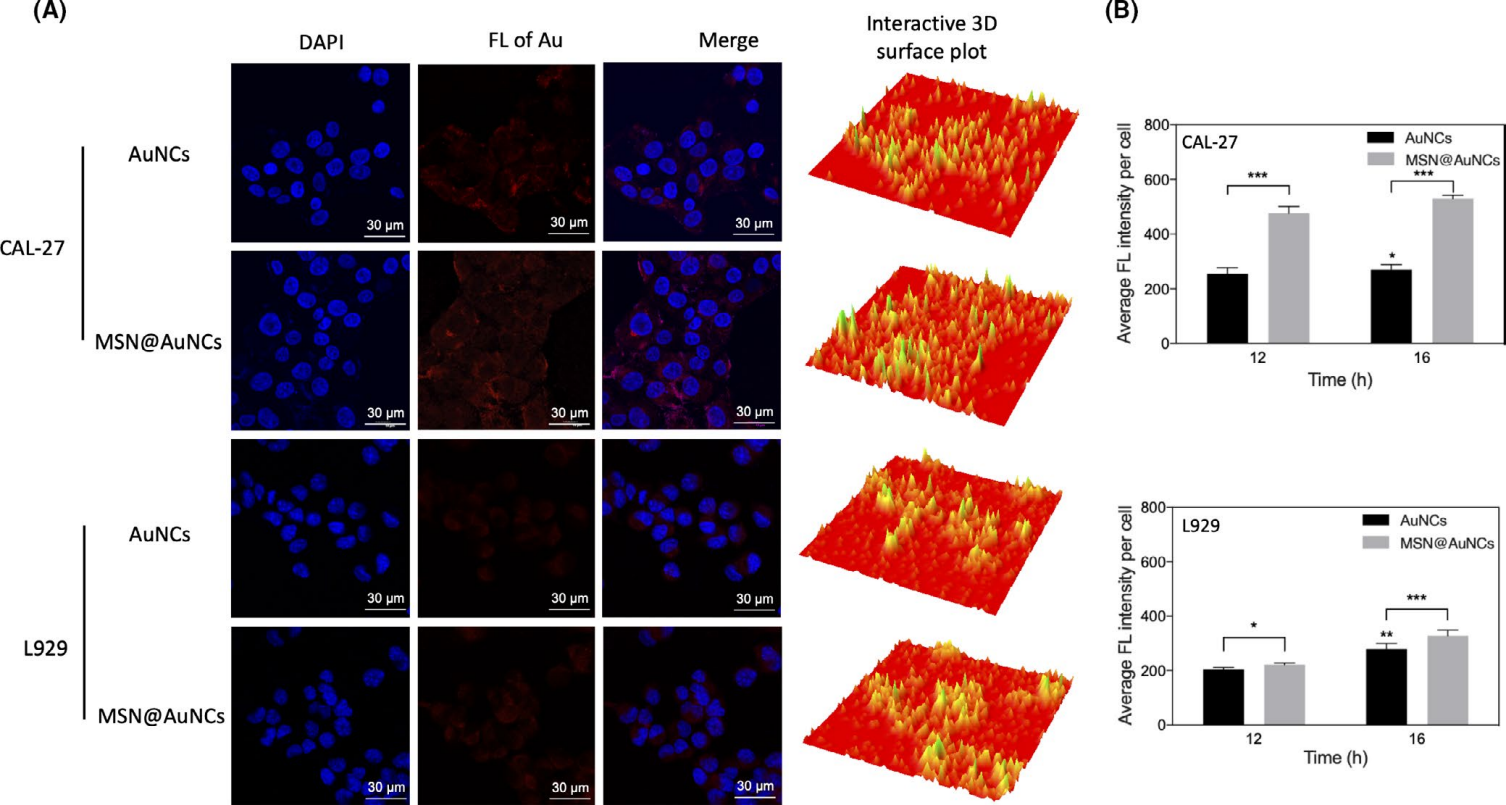
(A) Confocal laser scanning microscopy images and interactive 3D surface plots of CAL‐27 cells and L929 cells incubated with AuNCs and MSN@AuNCs for 6 hours; (B) average fluorescence intensity of CAL‐27 cells and L929 cells incubated with AuNCs and MSN@AuNCs for 12 and 16 hours by flow cytometry. Data are presented as mean ± SD (n = 3). *P < .05, **P < .005, ***P < .002

The fluorescence BSA‐AuNCs and MSN@AuNCs were prepared with the aim of comparing the feasibility of enhancing the fluorescence of CAL‐27 cells. Confocal microscopy analysis was used to observe the fluorescence intensity of the AuNCs and MSN@AuNCs. As exhibited in Figure 6A, after incubation for 6 hours, red‐emitted fluorescence was observed distinctly in the cytoplasm and membrane of CAL‐27 cells. Furthermore, the cells incubated with MSN@AuNCs emitted brighter fluorescence compared with those incubated in simple AuNCs, according to the confocal laser microscopy images and interactive 3D surface plots, which indicated that AuNCs enrichment could enhance the fluorescence imaging effect. In addition, L929 cells were measured under the same conditions. However, the fluorescence in the L929 cells was weak using both nanoparticles. A previous study showed that BSA is a functional protein that could stabilize nanomaterials and selectively recognize tumour cells.^32^ The capacity of BSA and the ability to absorb external substances of tumour cells led to the higher fluorescence intensity in CAL‐29 cells than in L929 cells. Therefore, enriching AuNCs into MSNs was feasible to enhance the fluorescence imaging effect in CAL‐27 cells.

To obtain the quantitative analysis results of the fluorescence intensity to further confirm the enhanced fluorescence of MSN@AuNCs in CAL‐27 cells, flow cytometry was performed. First, CAL‐27 cells and L929 cells were incubated with AuNCs and MSN@AuNCs for 12 hours and 16 hours, respectively. Then, the cells were excited at 355 nm by flow cytometry. As shown in Figure 6B, the average fluorescence intensity per cell of the CAL‐27 cells was about 255 and 476 after cultured with the AuNCs and MSN@AuNCs, respectively, for 12 hours. When incubated for 16 hours, the fluorescence intensity of the cells cultured in MSN@AuNCs increased to 529, which was much higher than that of cells cultured in AuNCs. Therefore, the MSN@AuNCs in CAL‐27 cells exhibited increased fluorescence brightness compared with that of the simple AuNCs. For L929 cells, the fluorescence intensity per cell was relatively weak and a significant difference could be observed between AuNCs and MSN@AuNCs after uptake for 16 hours.

## 4 DISCUSSION

In this study, nanoscale BSA‐AuNCs were prepared as a brown solution using a simple method. The red‐emitted fluorescence of BSA‐AuNCs was verified through UV‐vis absorption and 3D fluorescence spectra. Compared with traditional organic fluorescence dyes and fluorescence proteins, AuNCs, with low toxicity and excellent light stability, are more suitable as fluorescent imaging media for research. The synthetic MSNs possess a high surface area and uniform pore diameter. Carboxyl‐functionalized BSA‐AuNCs and amine‐functionalized MSNs were combined using electrostatic interactions and EDC/NHS coupling. The results of zeta potential and TEM showed that the MSN@AuNCs were prepared successfully. The enriched nanoparticles were non‐cytotoxic and biocompatible.

The characteristics of the AuNCs and MSN@AuNCs were compared using multiple detection methods and subsequent in vitro experiments, which demonstrated that AuNCs possessed increased location concentration and showed excellent photostability after loaded into MSNs. Besides, the large Stokes shift of the AuNCs ensured that the MSN@AuNCs displayed higher fluorescence brightness with no self‐absorption.^23^ Furthermore, the CT values of MSN@AuNCs were markedly increased, without changing the density of the AuNCs. The high CT signals of the MSN@AuNCs confirmed their application potential to enhance CT imaging. In the present study, L929 cells, CAL‐27 cells, ACC‐2 cells and SCC‐25 cells were chosen to measure the cytotoxicity of the AuNCs and MSN@AuNCs. L929 cells are normal cells and were used as the control group, while CAL‐27 cells, ACC‐2 cells and SCC‐25 cells are all oral carcinoma cell lines. The nanoparticles exhibited non‐toxicity to any of these cells; however, they tended to promote the proliferation in ACC‐2 cells and SCC‐25 cells, which was not the purpose of our study. Hence, the CAL‐27 oral carcinoma cells, which MSN@AuNCs had no effect on, were chosen for fluorescence imaging. The fluorescence imaging performance of the MSN@AuNCs in CAL‐27 cells was apparently better than that of the simple AuNCs, which supported the strategy of loading AuNCs into MSNs for enhanced fluorescence. In addition, the nanoparticles exhibited a higher cellular uptake in tumour cells than in normal cells. Therefore, compared with the simple AuNCs, MSN@AuNCs exhibited higher performance in both fluorescence and CT imaging. The above‐mentioned strategy successfully achieved improved NIR fluorescence/CT signals and revealed an important potential to create sensitive dual‐modal imaging probes.

In this work, MSN@AuNCs were prepared for enhanced NIR fluorescence/CT dual‐modal imaging, which improved each imaging function without complex modification, and provided a strategy reference for the construction of high brightness multimodal imaging probes in the future. To improve the targeting of this probe, MSN@AuNCs could be loaded with folic acid (FA), glutathione (GSH) or the transferrin receptor for tumour targeting in the future studies. High brightness NIR fluorescence/CT dual‐mode imaging probes can be used widely for tumour imaging analysis. Moreover, as a superior drug delivery medium, the imaging probes could be loaded with anti‐tumour drugs for tumour‐targeted treatment. The dual‐modal imaging probes are of great scientific significance to achieve high‐sensitivity live tumour multimode imaging, to construct novel drug delivery systems, and could promote the development of clinical cancer precision diagnosis and treatment.

## 5 CONCLUSIONS

In summary, multimodal imaging technology, achieved by integrating two or more imaging functions, can complement single‐mode imaging technology and obtain more information on lesions through the detection of multiple types of images, which is significant to promote the development of accurate clinical disease diagnosis.^33^, ^34^, ^35^ In our study, AuNCs with both fluorescence imaging and CT imaging functions were enriched into the non‐toxic biocompatible material MSNs using simple methods, which made up for the lack of spatial resolution in NIR fluorescence imaging and enhanced the imaging sensitivity of each mode, thereby obtaining high brightness fluorescence and CT imaging effects. We believe our dual‐modal imaging probes have a powerful potential for application in the diagnosis and treatment of disease.

## CONFLICT OF INTEREST

No conflict of interest was declared of this article.

## AUTHOR CONTRIBUTIONS

Yifang Yuan performed the experiments, analysed the data, constructed the figures and wrote the manuscript. Ronghui Zhou designed the experiments, constructed the figures and revised the article. Ting Li synthesized the nanoparticles. Shuang Qu and Hua Bai participated in experiments. Jiawu Liang drew the scheme pictures. Bin Guo and Cai Xiaoxiao contributed to conception and critically revised the manuscript. All authors gave their final approval and agreed to be accountable for all aspects of the work.

## Data Availability

The data, supporting the findings of this work, are available from the corresponding author upon reasonable request.
